# Multi-component quantitative magnetic resonance imaging by phasor representation

**DOI:** 10.1038/s41598-017-00864-8

**Published:** 2017-04-13

**Authors:** Frank J. Vergeldt, Alena Prusova, Farzad Fereidouni, Herbert van Amerongen, Henk Van As, Tom W. J. Scheenen, Arjen N. Bader

**Affiliations:** 1 Laboratory of Biophysics, Wageningen University & Research, Wageningen, The Netherlands; 2 Wageningen NMR Centre, Wageningen University & Research, Wageningen, The Netherlands; 3 Department of Pathology and Laboratory Medicine, UC Davis Medical Center, Sacramento, CA USA; 4 MicroSpectroscopy Centre, Wageningen University and Research, Wageningen, The Netherlands; 5 Department of Radiology and Nuclear Medicine, Radboud University Medical Centre, Nijmegen, The Netherlands

## Abstract

Quantitative magnetic resonance imaging (qMRI) is a versatile, non-destructive and non-invasive tool in life, material, and medical sciences. When multiple components contribute to the signal in a single pixel, however, it is difficult to quantify their individual contributions and characteristic parameters. Here we introduce the concept of phasor representation to qMRI to disentangle the signals from multiple components in imaging data. Plotting the phasors allowed for decomposition, unmixing, segmentation and quantification of our *in vivo* data from a plant stem, a human and mouse brain and a human prostate. In human brain images, we could identify 3 main *T*_*2*_ components and 3 apparent diffusion coefficients; in human prostate 5 main contributing spectral shapes were distinguished. The presented phasor analysis is model-free, fast and accurate. Moreover, we also show that it works for undersampled data.

## Introduction

Magnetic resonance imaging (MRI), like all other imaging modalities, thrive on our brains’ ability to interpret images in a very efficient way. This, in combination with experimentally encoding the magnetic resonance signal as a function of a wide range of material or tissue properties, explains the success of MRI in many disciplines of science and medicine, since its invention in the seventies^1, 2^.

A major benefit of MRI compared to other imaging modalities is that it provides excellent soft-tissue contrast over large anatomical areas, and additionally provides quantitative information on a number of magnetic resonance (MR) related parameters^3–6^. In quantitative MRI (qMRI), signals are not only encoded for space to construct an image, but also non-spatially to derive these quantitative parameters. The non-spatial information can consist of signal attenuation as a function of (i) inversion or saturation recovery time for longitudinal relaxation time *T*_*1*_, (ii) spin-echo time for transverse relaxation time *T*_*2*_, (iii) the *b*-value for diffusion mapping, or (iv) chemical shift information of different metabolites in an NMR spectrum^7^. These parameters depend on the local environment of the observed nucleus and enable for example the use of water as an intrinsic probe molecule.

In MRI, the signal from each data pixel can consist of an unknown number of different components. There is an increasing demand to retrieve quantitative information about the sub-pixel composition. In practice, however, this is often challenging because for *in vivo* studies on small animals or tissues at high spatial resolution the signal-to-noise ratio (SNR) is typically low, while clinical applications on humans at 1.5 and 3 Tesla are limited by total examination time and the maximum allowed deposition of radiofrequency power with the MR pulse sequence (the specific absorption rate limit, SAR). Common approaches to extract the quantitative parameters from the images are e.g. to fit multi-exponential decays or perform principle component analysis on spectra. It is often questionable whether there is sufficient SNR available to perform multi-component analysis. Moreover, most of these analysis algorithms require prior knowledge or model assumptions, which is inherently prone to systematic errors.

To overcome these issues in qMRI, we introduce the phasor approach: a toolbox that was earlier developed for the use in optical microscopy^8–11^. It is based on the principle that each harmonic of the discrete Fourier transform of a normalized exponential function or spectrum only depends on the shape of that function. Each harmonic forms an orthogonal set called a phasor. The phasors of all the pixels in the image can be visualized by plotting the imaginary vs. the real component of the first harmonic. As phasor coordinates are only dependent on the parameters that describe the shape of the function, the 2D phasor plot can be used to unravel the quantitative content in the image.

The application of this method in qMRI is illustrated with a multiple-spin-echo decay analysis for quantitative *T*_*2*_ mapping (*T*_*2*_-MRI) of a plant stem and the brain of a healthy human volunteer, with quantitative diffusion mapping of both a human and mouse brain, and with an analysis of spectroscopic imaging of the prostate of a patient with biopsy-proven prostate cancer. With these examples the underlying principles of the phasor approach can be understood and the most important features including quantification, segmentation and unmixing of multiple components become apparent. We will also show that undersampling of the non-spatial information is allowed for phasor based analyses. The key property that the phasor approach adds to existing analysis routines is that global component characteristics can be estimated from the phasor plot, meaning that it does not require prior knowledge of the sample composition. The subsequent unmixing is based on simple equations, so no iterative algorithms are applied.

## Results

**Figure 1:**
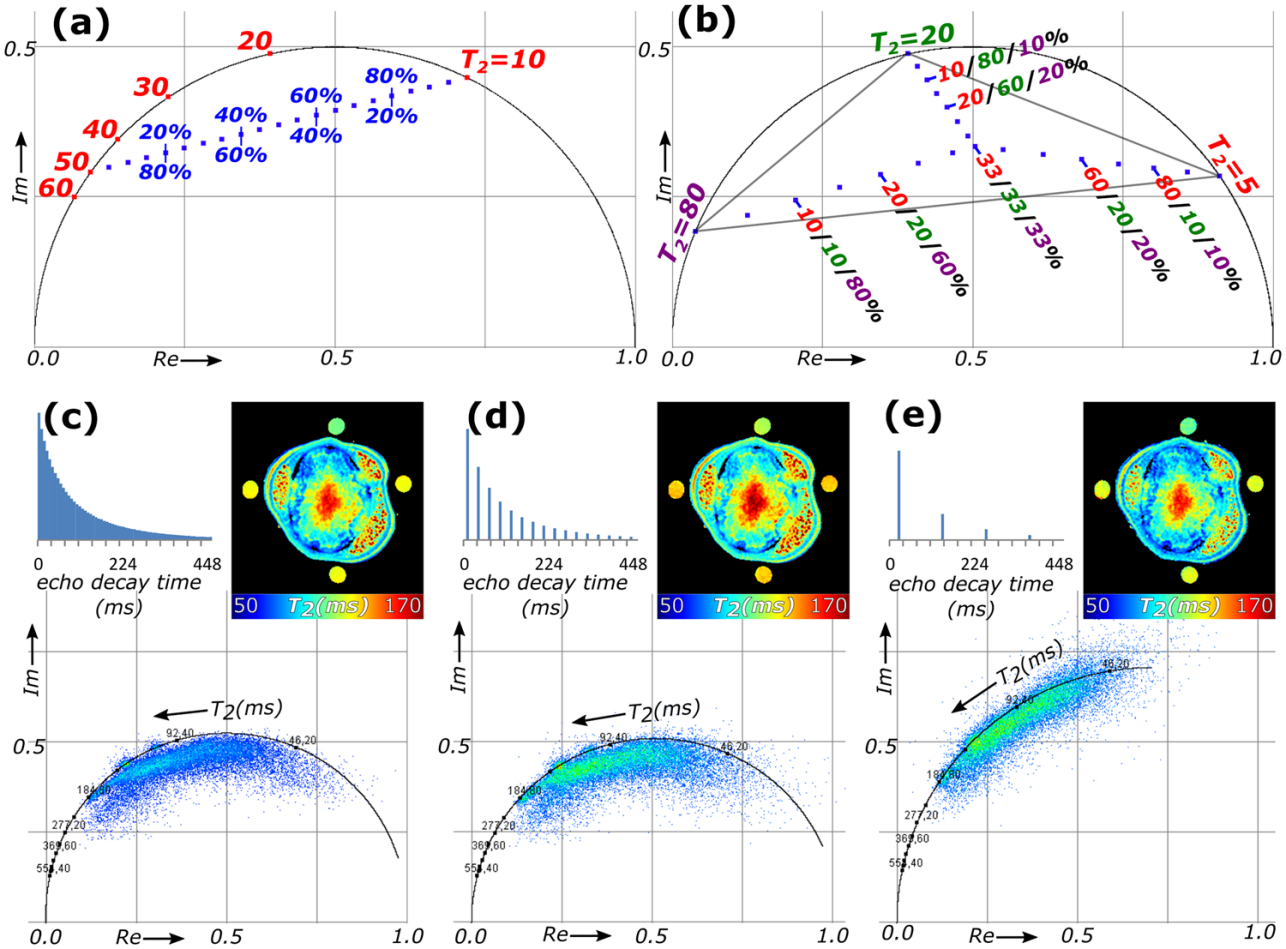
Concept of multi-component phasor analysis and the effect of undersampling. (**a**) For modelled datasets of mono-exponential decays with different time constants the phasors end up on the semicircle (red dots) between 0 for slow and 1 for fast decays, while for bi-exponential decays with different ratios of their total integral the phasors reside on a line (blue dots) between the phasors on the semicircle of the two mono-exponential decays comprising the signal. (**b**) For three components, the combined phasors are located inside a triangle connecting the three references. Comparison of (**c**) the phasor plot for the full 2D quantitative *T*_*2*_ dataset consisting of 64 echo time steps of the cross section of the stem of a tomato plant to (**d**) the phasor plot of a subset of 16 echo times with reduced SNR by extracting echo step 2, 6, 10, …, 62 from the full dataset and (**e**) the phasor plot of a subset of 4 echo times with further reduced SNR by extracting echo time steps 4, 20, 36 and 52 from the full dataset.

The concept of the phasor approach for qMRI is first demonstrated with noise-free, modelled multi-echo relaxation curves simulating MR signal attenuation decay due to *T*_*2*_ relaxation. Each decay is (1) normalized, (2) Fourier transformed and (3) plotted as a phasor with coordinates (Re_n=1_, Im_n=1_), where n = 1 refers to the first harmonic of the Fourier transform. Mono-exponential decays are located on a semicircle in a phasor plot (Fig. 1a). The phasor of a bi-exponential decay is a linear combination of the phasors of the mono-exponential references, i.e. the two reference phasors are added up like vectors. Because the magnitudes of the reference phasors are shortened to their fractional contributions, the phasor of a bi-exponential decay is located on a straight line connecting the phasors of the individual components on the semicircle (Fig. 1a). In the same way, mixtures of three decays are located inside a triangle connecting the individual components on the semicircle (Fig. 1b). The phasor coordinates (Re_n=1_, Im_n=1_) can also be used to quantify the average *T*_*2*_ in each pixel (see Supplementary Information for more details)^12^.

The use of the phasor approach was tested with a *T*_*2*_-MRI measurement of the cross section of a plant stem (Fig. 1c–e). For this sample, we were not limited by SAR or examination time constraints and therefore SNR could be maximized. Structures that could be distinguished in the image were the epidermis and cortex on the outside, three areas of vascular tissue (containing phloem and xylem), and the pith in the center. The stem was surrounded by four reference tubes. The *T*_*2*_-MRI dataset contained exponential signal decays of 64 echo times for each pixel. For this dataset, the phasor plot was calculated (Fig. 1c); an extended cloud of phasors was located inside the semicircle. The insert shows the average *T*_*2*_ map calculated from the phasors.

To reduce measurement time, undersampling is used in many applications of qMRI. One way to achieve this is by reducing the number of echoes to e.g. 16 or 4, as shown in Fig. 1d and e, respectively. Compared to the 64 echo image, the low number of echo time steps and the truncation of the exponential decay resulted in a deviated semicircle of mono-exponential reference decays in the phasor plots and consequently an alternative form of Supplementary Equation 6 had to be applied to calculate the average *T*_*2*_^12^ (see Supplementary Information for details). The cloud of phasors retained its shape and location inside the new semicircle. As undersampling was achieved by discarding echoes, the total SNR decreased, which resulted in more scatter in the phasor plot. The average *T*_*2*_ maps were not notably affected by the lower number of echo times and lower signal. This demonstrates that a phasor analysis can be used for average *T*_*2*_-quantification also for undersampled datasets.

**Figure 2:**
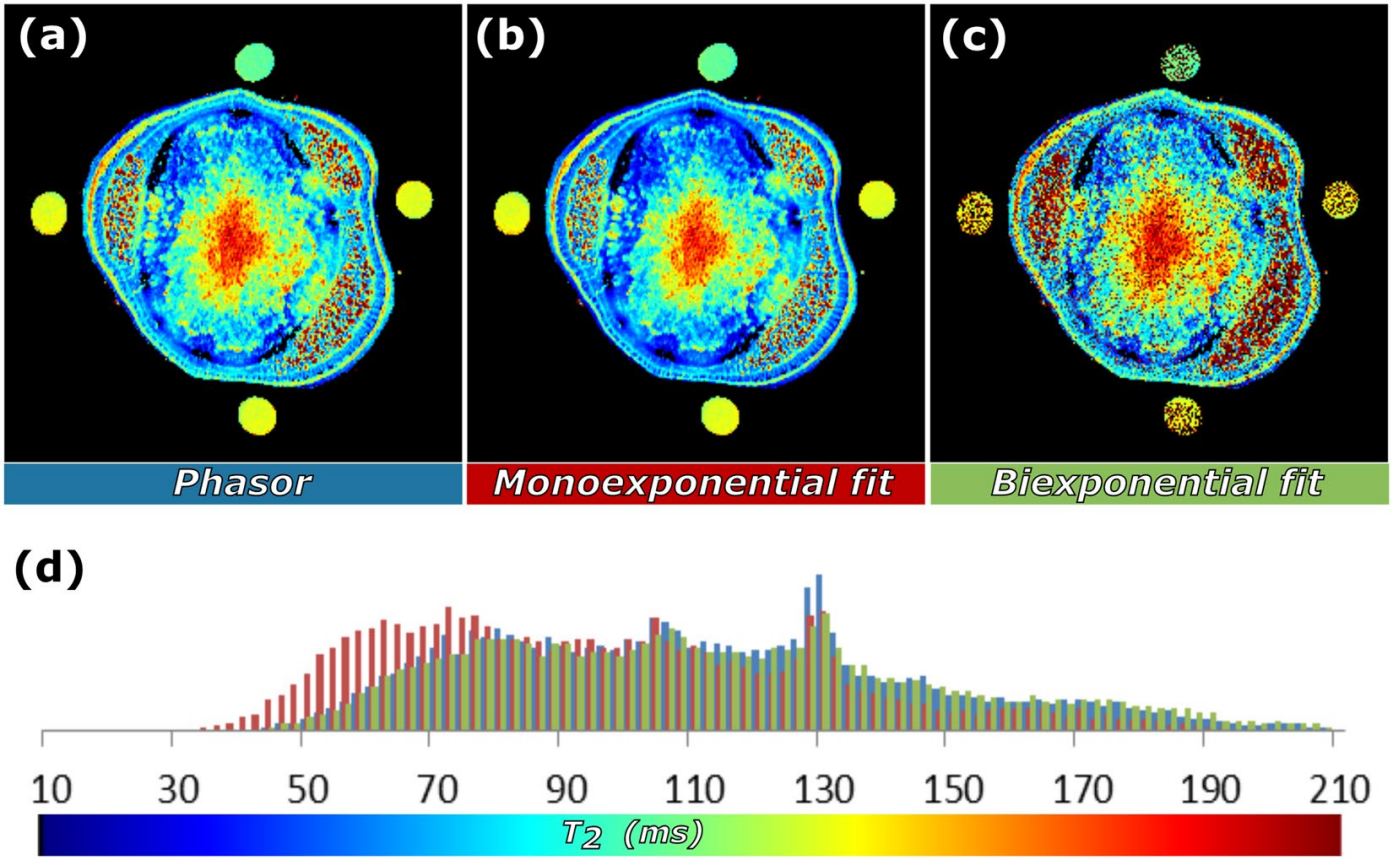
Comparison of quantification by phasor analysis and the Levenberg-Marquardt decay fitting algorithm. *T*_*2*_ maps of a stem of a tomato plant based on (**a**) phasor analysis, (**b**) mono-exponential and (**c**) bi-exponential fitting; (**d**) comparison of the *T*_*2*_ histograms for the three methods of quantification.

To validate the outcome of the phasor analysis, the results were compared to a general accepted fitting procedure for exponential decays (Levenberg-Marquardt algorithm, see Fig. 2): the *T*_*2*_ maps based on mono-exponential and bi-exponential (the weighted average of two *T*_*2*_’s) fitting routines are shown in Fig. 2b and c, respectively. There was good agreement between the calculated *T*_*2*_ values obtained by phasor (Fig. 2a) and exponential fitting. The phasor plot provided evidence that most of the individual pixel decays were multi-exponential since their phasors were located inside the semicircle (Fig. 1c). Analyzing this dataset with mono-exponential decays resulted in a bias of the *T*_*2*_ values; they were shorter than those obtained with phasor analysis (Fig. 2d). Bi-exponential fitting, however, gave much more misfits (5% of the thresholded pixels gave unrealistic *T*_*2*_’*s* or could not be fitted) and poor results for the four reference tubes surrounding the plant stem (these should have mono-exponential decays and can be distinguished in the phasor plot as circular clouds on the semicircle at 105 and 130 ms, see Fig. 1c). This illustrates that the phasor approach gives a result that is similar to exponential decay fitting, but is independent of the number of components per pixel.

**Figure 3:**
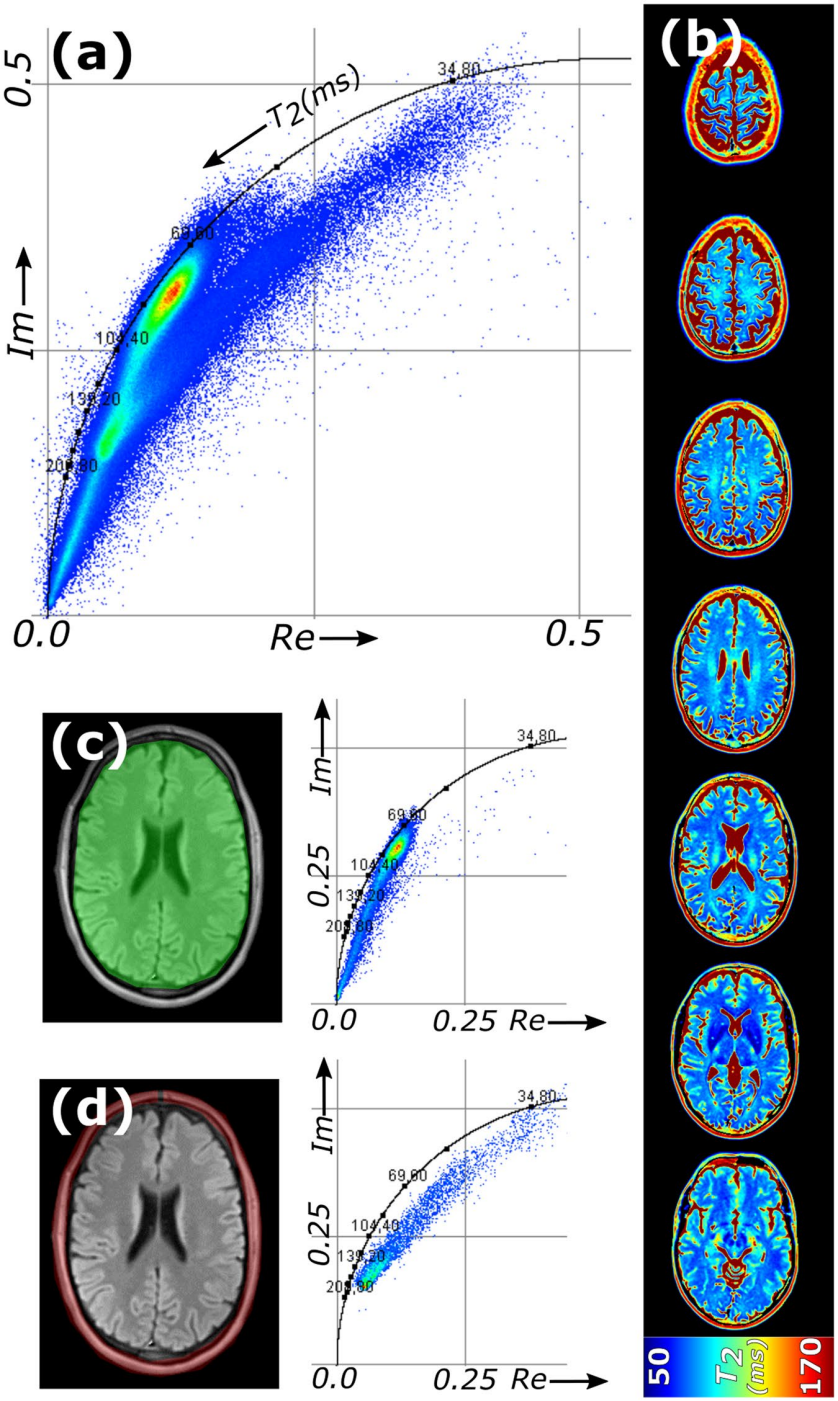
Phasor analysis of a quantitative *T*_*2*_ MRI dataset of an *in vivo* human head. (**a**) Phasor plot of a multislice 2D quantitative *T*_*2*_ dataset of a human brain (10 echoes); (**b**) *T*_*2*_ maps of a representative subset of slices (full set available as Supplementary Video 1); spatial segmentation followed by phasor analysis of (**c**) the brain tissue and (**d**) the skull.

Our next analysis involved a multi slice quantitative *T*_*2*_ MRI measurement of the head of a healthy human volunteer (10 equidistant echo times). The shape of the cloud in the phasor plot (Fig. 3a) gave information about the distribution of *T*_*2*_ times in the images, the average *T*_*2*_’s were quantitatively mapped in Fig. 3b. In the phasor plot multiple, partly overlapping clouds could be recognized. By performing phasor analysis on selected regions in the image, one cloud originating from the brain (Fig. 3c) and one from the small lipid layer around the skull (Fig. 3d) could be distinguished. For the brain, the main phasor cloud has an oval shape with average *T*_*2*_’s between 70 and 90 ms and is located inside the semicircle, indicating multi-exponential decays in most brain cells. From the 90 ms edge of the main cloud, there is a tail towards the origin of the phasor plot. This tail contains the phasors with relatively long average *T*_*2*_, and originates from the pixels on the edges of the brain. The tail does not follow the semicircle but is a straight line instead, which suggests that there is a group of pixels with bi-exponential decays with fixed *T*_*2*_’s but varying fractional contributions. The reference *T*_*2*_’s can be estimated by extrapolation of the cloud. From this analysis, we found that the pixels on the edge of the brain and contain both intracellular water (80 ms) and cerebrospinal fluid (CSF, *T*_*2*_ > 500 ms) in varying ratio. For lipids around the skull, there is also a linear combination of two *T*_*2*_ times with variable contributions (Fig. 3d): a long *T*_*2*_ = 210 ms (water) and a short *T*_*2*_ = 30–40 ms (lipids). The estimation of the reference *T*_*2*_’s was guided by the shape of the phasor cloud; no prior knowledge about *T*_*2*_’s in brain tissue was needed to perform the analysis.

**Figure 4:**
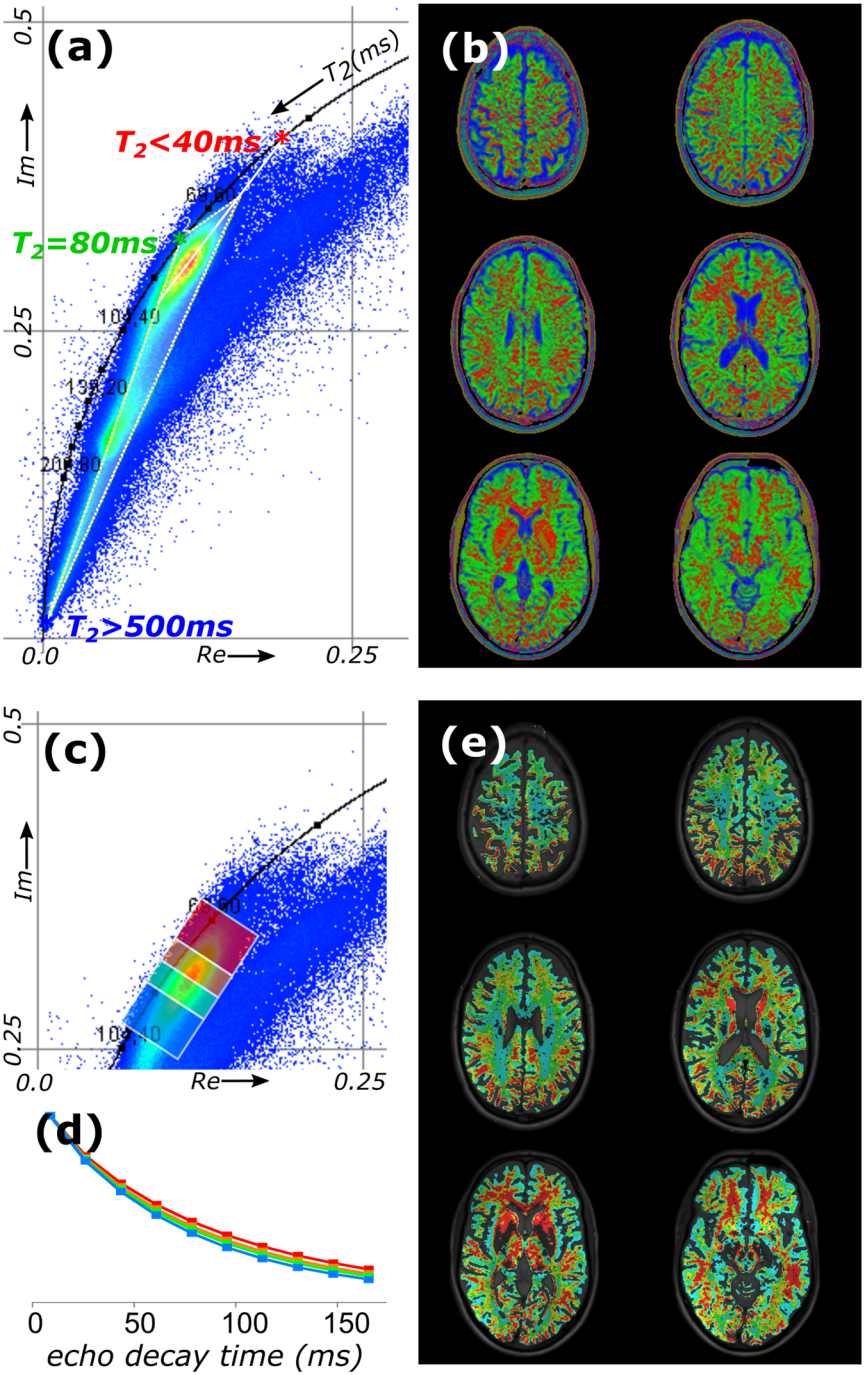
Multicomponent *T*_*2*_ phasor analysis of an *in vivo* human head. (**a**) Unmixing of the phasors into three mono-exponential *T*_*2*_ attenuation decays on the semicircle resulting in (**b**) RGB contribution maps of the separate components (full set available as Supplementary Video 2). The three components are *T*_*2*_ = 40 ms (blue), 80 ms (green) and >500 ms (red). For segmentation, four regions are selected in the phasor plot (**c**), color coded red, yellow, green and blue; (**d**) average *T*_*2*_ decays for the segmented regions, and (**e**) color-coded back-projection of the segmented pixels into the images (full set available as Supplementary Video 3).

From the phasor cloud, the *T*_*2*_ values of a third main component in brain tissue could be extracted. Besides the cloud of pixels that is located on a line connecting *T*_*2*_ > 500 ms and *T*_*2*_ = 80 ms (Fig. 4a), the major phasor cloud of brain tissue extends towards a shorter *T*_*2*_ value of approximately 40 ms. These three *T*_*2*_ values correspond to values reported elsewhere^4, 13–15^. Notably, the short *T*_*2*_ between 10 and 50 ms in brain originates from water trapped between the bilayers of the myelin sheath^13, 16^, which is predominantly found in white matter. An additional feature of phasor plots is that they can be used to unmix three components^11^. This inmixing procedure is non-iterative and based on a single equation for each component that divides the area of a triangle of the phasor and the two opposing reference phasors by the area of the reference triangle (see Fereidouni *et al*.^11^ for more details). It therefore calculates the fractional contribution of the three component (here *T*_*2*_ = 40, 80 and 500+ ms) in each pixels, which was displayed as respectively R, G and B color in the image (Fig. 4b). The reference phasor for the red channel was shifted towards the center of the main phasor cloud for better visualization. Overall, the phasor approach unmixed the three main pools: the blue channel maps CSF, the red channel corresponds to myelin water in white matter and the remaining green channel is brain tissue (gray matter).

To distinguish more components, the phasor approach can segment pixels based on phasor coordinates. As an example, in the oval-shaped main peak (*T*_*2*_ = 70–90 ms) four regions of interest were hand drawn, which segments pixels with similar parametric composition (Fig. 4c). The signal decay curves were averaged over each region and plotted in Fig. 4d. Moreover, the phasors in these regions were color-coded and back-projected into the image (Fig. 4e). The color-coding highlights symmetrically grouped regions in the brain with the same *T*_*2*_, and helped identify small differences in *T*_*2*_ in the brain tissue, related to the brain’s morphology^17^. Notably, the pixels were segmented based on similarities in their *T*_*2*_ decays, without the need to quantify their *T*_*2*_ values.

**Figure 5:**
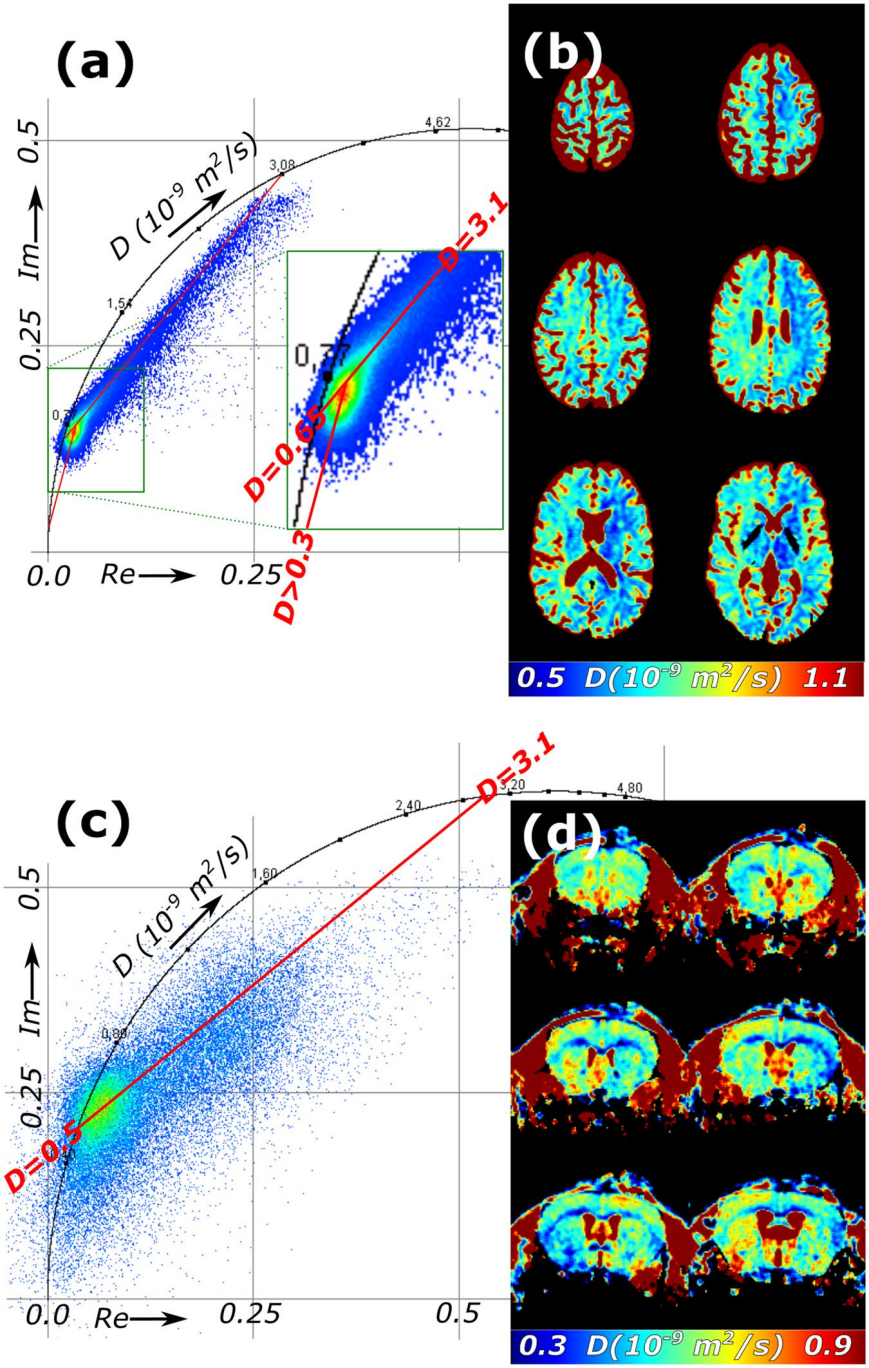
Phasor analysis of multislice 2D quantitative diffusion MRI datasets of *in vivo* human and mouse head. Datasets containing (**a**,**b**) 13 b-values per decay of a human and (**c**,**d**) 5 b-values per decay of mouse brain gives (**a**,**c**) phasor plots and (**b**,**d**) average apparent diffusion coefficient maps of representative subsets of slices (full set available as Supplementary Videos 4 and 5). The red lines in the phasor plots are the extrapolations of the phasor cloud that are used to estimate the ADCs of the components.

qMRI datasets for diffusion mapping are very similar to datasets for *T*_*2*_ mapping: there is a signal attenuation in each pixel of the image. Rather than echo time dependent, the MR signal is a function of duration, amplitude and spacing of pulsed field gradients^18^, summarized in the *b*-value^19^; the magnetization decays exponentially with this experimental parameter and a decay constant that is equal to diffusion coefficient *D* (see Supplementary Information for more details). The phasor plots of multi slice two-dimensional diffusion MRI datasets with 13 *b*-values of *in vivo* human and 5 *b*-values for *in vivo* mouse head showed a cloud of phasors inside the semi-circle (Fig. 5a and c) and a line of points extending to longer apparent diffusion constant (*ADC*) (Fig. 5a). A map of the average *ADC* is shown in Fig. 5b for human and Fig. 5d for mouse head.

In the phasor plot of diffusion MRI of a human head (Fig. 5a), most phasors had an average *ADC* of 0.70–0.75 × 10^−9^ m^2^/s, which is in good agreement with the *ADC* of restricted water in healthy brain^20^. Additionally, there was a linear phasor cloud that extended towards higher *ADC*s (3.1 × 10^−9^ m^2^/s), which is in good agreement with the *ADC* in CSF. More in depth analysis of the main phasor cloud (insert in Fig. 5a) was used to solve partial volume effects. Beside CSF, extrapolating the phasor cloud suggested that brain tissue has an *ADC* of 0.65 × 10^−9^ m^2^/s and part of the tissue has a third *ADC* (<0.3 × 10^−9^ m^2^/s). This latter contribution could originate from additional myelin in white matter, which is known to reduce the average *ADC* from 0.75 × 10^−9^ m^2^/s (deep grey matter) to 0.70 × 10^−9^ m^2^/s^21^. For mouse head imaging, the pixels were a lot smaller, and the SNR was lower. Still, an estimate of the main *ADC* components could be made (Fig. 5c and d): 0.5 × 10^−9^ m^2^/s for brain tissue and 3.1 × 10^−9^ m^2^/s for CSF. Notably, the *ADC*s of brain tissue was significantly lower for the mouse, but apart from that the shapes of the phasor clouds in Fig. 5a and c are similar.

**Figure 6:**
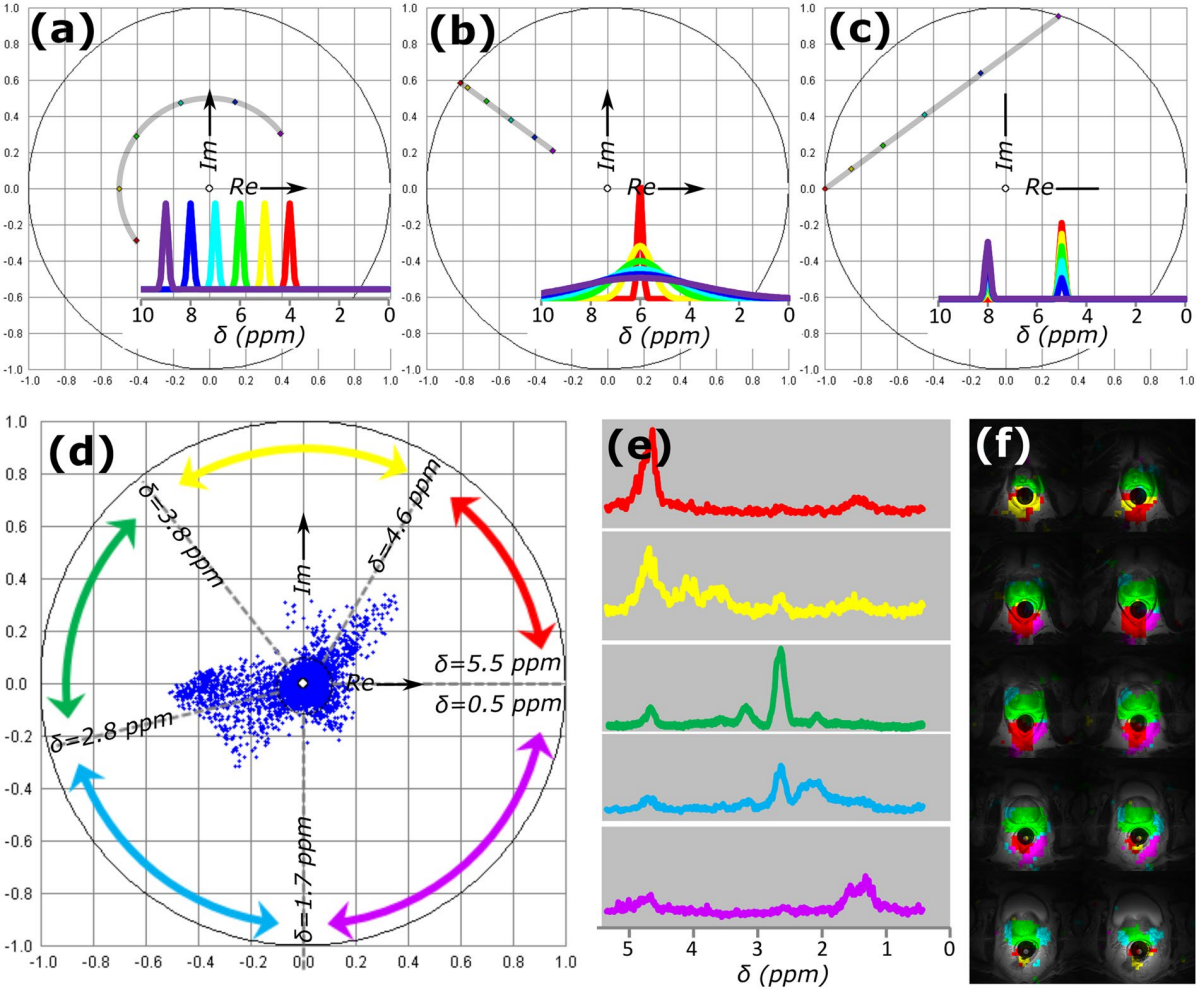
Phasor analysis of MRSI of the *in vivo* human prostate. For modeled datasets, (**a**) the phasors for Gaussian line shapes in a spectrum are determined by the position of the center of the peak on the spectral axis; peak shifts towards larger ppm values correspond to a clockwise direction on the phasor plot; (**b**) the phasor points for Gaussians on the same center position with different linewidths, narrow lines appear closer to the circle, the origin of the phasor plot is a flat baseline (infinitely wide line); (**c**) the phasors for spectra with multiple signals form a line between the phasors for the separate signals comprising the spectrum. For a 3D MRSI dataset of a human prostate with biopsy-proven prostate cancer (**d**) the phasor plot is segmented guided by the typical features of the plot; (**e**) average spectra of segmented pixels and (**f**) color coded back-projection overlaid with the *T*_*2*_-weighted MR images of the dataset.

To demonstrate spectral phasor analysis for MR spectroscopic imaging (MRSI), we first analyzed synthetic NMR spectra (Fig. 6). In the phasor plot, spectra were segmented based on the chemical shift and the linewidth of the signal. Resonances with equal shape but increasing chemical shift moved clockwise on a circle (equal distance from phasor origin, Fig. 6a). The distance to the center was determined by the linewidth of the resonance, ranging from 1 for infinitely narrow linewidths to the origin of the phasor plot for a flat baseline (Fig. 6b). MR spectra are typically composed of multiple signals, so the resulting phasor will be a linear combination of the phasors of its individual signals (Fig. 6c).

The spectral phasor analysis was applied to an MRSI dataset of the prostate of a patient with biopsy-proven prostate cancer (Fig. 6d–f). NMR spectra were spatially resolved with low resolution in a three-dimensional MRSI measurement, which was overlaid onto a high spatial resolution *T*_*2*_-weighted MRI dataset. The chemical shift of interest for ^1^H spectroscopy ranged from 0.4 to 5.4 ppm, with prostate-specific metabolites (citrate, choline, creatine and polyamines) at shifts of 2.5 to 3.5 ppm. Many of the spectra in the MRSI dataset had flat noisy baselines with no signal due to its distance from the endorectal receive coil, resulting in phasors close to the origin. Five small clouds protruded indicating that these pixels contained additional spectral features. The phasor plot was divided in five regions (Fig. 6d) and the pixels of the five clouds were segmented. The average spectra in the segmented regions contained the respective signals of interest in the corresponding chemical shift regions (Fig. 6e). The selected pixels were back-projected onto the high resolution *T*_*2*_-weighted images (Fig. 6f). The red and yellow pixels contain residual water signal (4.7 ppm) and are located outside of the prostate (peri-rectally and around the left neurovascular bundle). The peri-rectal purple pixels had some residual lipid signal. Signals of interest (from 2.5 to 3.3 ppm) were mainly present in the green region, covering the complete prostate. Despite the complexity of this *in vivo* data sample^22^, phasor analysis could disentangle its chemical composition.

## Discussion

Imaging technologies have made their way as essential tools in modern science. Different imaging modalities generate contrast in their own specific way. In fluorescence lifetime imaging microscopy, the nanosecond decay of fluorescent tags attached to e.g. proteins in cell systems is recorded for each pixel in an image. The complexity in the analysis of this kind of data had been overcome by the phasor approach^23–25^. We introduced in this work its use for qMRI, but it can be used in other imaging techniques as well.

In qMRI, the phasor approach is an alternative to iterative fitting algorithms (typically Levenberg-Marquard algorithm) of the characteristic single-pixel signal attenuation curves. As shown in Fig. 2, this yields an almost identical distribution of average decay constants; the histograms of the obtained decay times strongly overlap. Compared to fitting the phasor approach is faster and independent on initial guesses. It provides an (weighted) average decay constant independent of the number of exponents in the decay.

In a phasor plot, pixels are grouped by their non-spatial, quantitative properties and characterized by means of the multi-component composition they share with other pixels. Regardless of location in the image, pixels with similar properties end up at the same position in the phasor plot, allowing recognition of exact quantitative values, and allowing backprojection of pixels with the same properties. More importantly, if multiple pixels share components in varying amounts, extrapolating the phasor cloud to the reference semicircle can be used to find the characteristic properties of the contributors. We showed here that for both quantitative *T*_*2*_ mapping and in diffusion MRI of healthy brain we could disentangle 3 main components. Components were mathematically unmixed, and individual components could be mapped.

For MR spectroscopic imaging, the same tools can be applied although the signals that were transformed into phasors were completely different. The spectral linewidths of MR signals in MRSI were much narrower than those in fluorescence spectral imaging^11^, but also for narrow Raman bands the phasor analysis has recently proven to be useful^26^. As such, it can be applied to unravel the composition of complex samples, or filter out fractions that are not of interest. Here, the spectral phasor was used to identify five main components in a human prostate.

On the long term, the phasor approach could also be used in clinical applications. It displays deviations in quantitative parameters and could therefore identify abnormalities that may relate to a pathology, the phasor plot has the potential to be used for diagnostic purposes. It is based on simple mathematics so it is fast, robust, reproducible, and quantitative, allowing automated signal processing systems. The phasor approach also works for undersampled data and can therefore contribute to the ongoing effort to speed up MRI experiments by many modern concepts in acquisition, post-processing and visualization, like MR fingerprinting^27, 28^, parallel imaging^29, 30^, sparse sampling^31^, and computer aided analysis.

## Methods

### MR imaging

The *T*_*2*_-MRI experiment of the cross section of the main stem of a tomato plant (*Solanum lycopersicum* L.) was measured with a multi spin echo imaging sequence^32^ on a dedicated vertical 3 T MRI-spectrometer^33^ operated by a Bruker Avance console with ParaVision 3 software (Bruker BioSpin, Karlsruhe, Germany). In total 128 images with an inter-echo spacing (*TE*) of 7.23 ms were recorded. The first 64 echo steps were used for analysis. The images of 256 × 256 pixels were recorded with a field of view (*FOV*) of 22 × 22 mm^2^ and a slice thickness of 1.5 mm. The repetition time (*TR*) was 6 s and the number of averages (*NA*) 16, resulting in a total acquisition time (*TA*) of almost 7 hours. To demonstrate the effect of undersampling in Fig. 1d and e, 64 echoes were reduced to 16 and 4 echoes by extracting subsets from the full echo train.

Multislice 2D *T*_*2*_-MRI and diffusion weighted images of the head of a healthy human volunteer were acquired on a 3 T whole body MR system (Magnetom TimTrio, Siemens Healthcare, Erlangen, Germany). Examination parameters *T*_*2*_-MRI: 20 echoes with *TE* 8.7 ms, *FOV* 220 × 220 mm, 256 × 256 pixels, 15 slices, slice thickness 3 mm, inter-slice gap 3 mm, *TR* 3 s, *NA* 1, and *TA* 7 min 58 s. Examination parameters diffusion mapping: echo planar imaging of a single spin echo, *TE* 67 ms, *FOV* 179 × 220 mm^2^, 130 × 160 pixels, 15 slices, slice thickness 3 mm, inter-slice gap 3 mm, *TR* 2.5 s, parallel imaging acceleration factor 3, *NA* 1, *TA* 6 min 23 s, and 3 orthogonal diffusion directions with 13 *b*-values (0, 300–1200 s/mm^2^).

2D diffusion weighted images of a mouse brain were acquired on a 11.7 T animal MR system (BioSpec 117/16, Bruker BioSpin HmbH, Reinstetten, Germany). Examination parameters: imaging of a single spin echo, *TE* 27 ms, *FOV* 12.5 × 12.5 mm^2^, 128 × 128 pixels, 1 slice, slice thickness 0.5 mm, *TR* 2.25 s, *NA* 1, *TA* 24 min, and 3 orthogonal diffusion directions with 5 *b*-values 0, 500–2000 s/mm^2^.

Multislice 2D magnetic resonance spectroscopic imaging of the prostate was performed on a patient with biopsy-proven prostate cancer on the same 3 T whole body MR system with an endorectal receive coil. Examination parameters: dual-frequency selective suppression of water and lipids, *TE* 145 ms, *FOV* 84 × 60 × 72 mm, 14 × 10 × 12 pixels, *TR* 750 ms, weighted elliptical sampling with *NA* 3, and *TA* 8 min 19 s.

All experiments were carried out in accordance with relevant guidelines and regulations. The MRI examination of the healthy volunteer was approved by the local institutional review board (METC CMO Regio Arnhem-Nijmegen) and the volunteer gave written informed consent prior to the MR exam. The animal experiment was performed according to the Dutch federal regulations for animal protection and approved by the Veterinary Authority of Radboud University Medical Center, Nijmegen, The Netherlands and the Animal Experiment Committee of the Radboud University, Nijmegen, The Netherlands.

### Phasor analysis

The MRI images were imported as raw data in ImageJ/FIJI, with time/*b*-value/chemical shift as a third axis. For phasor analysis, minor modifications were made to the time-gated-phasor and spectral-phasor plugins. The plugin returns the phasor plot and *T*_*2*_ map; for diffusion MRI the plugin was converted to make ADC maps. ImageJ has built-in functions to generate histograms, change lookup tabel and convert images to TIF. The plugin also provides the phasor-unmix function that was used to make Fig. 4b; the three references were manually added. Backprojection of selected phasors (Figs 4c–e and 6d–f) was performed by drawing polygon selections in the phasor plot and subsequently use the phasor-to-image function of the plugin; the resulting segmented images were color coded and combined, the spectra were copied to Excel for plotting. For Figs 1, 3–6, a mean filter with radius 1 was applied to each echo/*b*-step/chemical shift.

Mono/bi-exponential fitting of Fig. 2b–d was performed with Levenberg-Marquardt non-linear least squares algorithm implemented in the SplMod package^34^ on the same dataset as in Fig. 1c and with the same threshold. The *T*_*2*_ maps generated by SplMod were loaded into ImageJ to apply the same loop-up table and scaling. For bi-exponential decays, the weighted average *T*_*2*_ was plotted.

## Electronic supplementary material

Supplementary Information

Supplementary Video 1

Supplementary Video 2

Supplementary Video 3

Supplementary Video 4

Supplementary Video 5
