# Supplementary material for: Multi-component quantitative magnetic resonance imaging by phasor representation
Source: Sci Rep. 2017 Apr 13;7:861. doi: 10.1038/s41598-017-00864-8 (PMC5429833; doi:10.1038/s41598-017-00864-8)
Supplement: Supplementary file 1 — Supplementary Information [file 41598_2017_864_MOESM1_ESM.pdf]

Supplementary information of

Multi-component quantitative magnetic resonance imaging by phasor representation

Frank J. Vergeldt[1,2], Alena Prusova[1], Farzad Fereidouni[3], Herbert van Amerongen[1,4], Henk Van As[1,2*], Tom W. J. Scheenen[5], Arjen N. Bader[1,4*]

1. Laboratory of Biophysics, Wageningen University, Wageningen, The Netherlands
2. Wageningen NMR Centre, Wageningen University, Wageningen, The Netherlands
3. Department of Pathology and Laboratory Medicine, UC Davis Medical Center, Sacramento, CA, USA
4. MicroSpectroscopy Centre, Wageningen University, Wageningen, The Netherlands
5. Department of Radiology and Nuclear Medicine, Radboud University Medical Centre, Nijmegen, The Netherlands

When recorded with a low number of points (i.e. echoes or *b*-values), a decay is undersampled and truncated. This means that Equations 1 and 2 are no longer valid. A modified phasor approach [12] can calculate the average decay constant (i.e. T_2 or $1/D$). For temporal decays *f*, which are sampled with total number of sampling of K and total measurement time window of T the signal is recorded in sampling intervals of T/K at integer multiples of *k*:

$$f(k) = e^{-\frac{(k+\frac{1}{2})\frac{T}{K}}{T_2}}$$

Figure S1 shows the schematic diagram for acquisition of data and used nomenclatures.

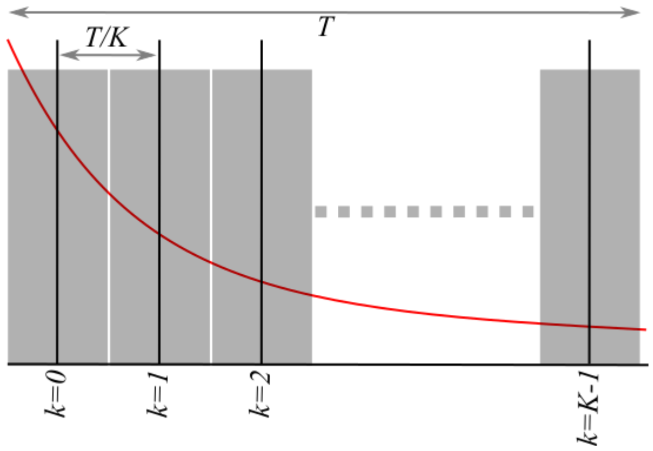

Fig. S1. Schematic diagram of the undersampling of a decay at intervals of T/K.

The normalized Fourier transformation of *f(k)* at first harmonics:

$$F(T_2) = \left[\sum_{k=0}^{K-1} e^{i\frac{2\pi}{T}(k+\frac{1}{2})\frac{T}{K}} \cdot e^{-\frac{(k+\frac{1}{2})\frac{T}{K}}{T_2}}\right] \Big/ \left[\sum_{k=0}^{K-1} e^{-\frac{(k+\frac{1}{2})\frac{T}{K}}{T_2}}\right]$$

Analytical solution of equation above, provides the exactly similar equation used to analyze binned fluorescence decay curves [12] :

$$F(T_2) = \frac{1}{\cos\left(\frac{\pi}{K}\right) - \sin\left(\frac{\pi}{K}\right)\coth\left(\frac{T}{2KT_2}\right) i}$$

T_2 can be estimated by using the real and imaginary part of $F(T_2)$:

$$T_2 = \frac{T}{2K\mathrm{arccoth}\left(\frac{\mathrm{Im}(F)}{\mathrm{Re}(F)}\cot\left(\frac{\pi}{K}\right)\right)}$$

When the sampling intervals is large, $T/K \to 0$ and

$$F(T_2) = \frac{1}{1 - \mathrm{i}\frac{2\pi}{T}T_2}$$

and T_2 can be estimated by:

$$T_2 = \frac{T}{2\pi}\frac{\mathrm{Im}(F)}{\mathrm{Re}(F)}$$

In a similar way, for diffusion MRI the magnetization decays exponentially with an experimental *b*-value ($M = M_0 e^{-bD}$ for mono-exponential decay, with diffusion coefficient *D*). For exponential decays composed of a large number of *b*-values, the average *ADC* can be estimated by:

$$ADC = \frac{2\pi}{B}\frac{\mathrm{Re}_{\mathrm{n=1}}}{\mathrm{Im}_{\mathrm{n=1}}}$$

To standardize and optimize the graphical representation of the phasors, we add ½ to *k*. For T_2, this means that the echo decay time axis is shifted by -½ echo time, while for diffusion MRI the *b*-axis is shifted by +½ '*b*-step'. These shifts only result in phase shifts in the Fourier domain, which is apparent as a rotation in the phasor plot. As shown above, the calculation of MRI parameters from phasor coordinates also takes this phase shift into account.

Supplementary video captions

Supplementary Video 1: T_2 maps of the full quantitative MRI dataset of an in vivo human head (figure 3B).

Supplementary Video 2: RGB contribution maps of the separate components in the quantitative MRI dataset of an in vivo human head (figure 4B).

Supplementary Video 3: Color-coded back-projection of the segmented pixels into the images of the quantitative MRI dataset of an in vivo human head (figure 4E).

Supplementary Video 4: Average apparent diffusion coefficient maps of a multislice 2D quantitative diffusion MRI dataset of an in vivo human head (figure 5B).

Supplementary Video 5: Average apparent diffusion coefficient maps of a multislice 2D quantitative diffusion MRI datasets of an in vivo mouse head (figure 5D).
